# Medical Items and News of Interest to the Physician

**Published:** 1878-10

**Authors:** 


### For the Use of Physicians.

CULLED FROM THE STANDARD MEDICAL JOURNALS OF THE WORLD.

NOTE.—These formulæ are intended only for the regular practitioner of medicine, and should be employed only by him, or under his immediate supervision.

**Freckles.**

Sulphocarbolate of zinc, 1 drachm.
Oil of lemon, 1 "
Alcohol, 5 drachms.
Collodion, 45 "

To be applied with a camel hair brush.

**Thiersch's Salicylic Mixture.**

Salycylic acid, 5 grammes,
Rect. spirit, 95 "
Water, 140 "
Syr. of orange peel, 60 "

Dose, a teaspoonful.

**Hair Tonic.**

Glycerine, 4 fluid ounces.
Tincture of cantharides, 5 fluid drachms.
Ammonia, 4 " "
Rose water, 3 " "
Bay rum, 10½ " ounces.

**Cod-Liver Oil with Chloral.**

The following mixture of cod-liver oil and chloral is said to effectively stop the night-sweats of consumptives, while at the same time producing sleep and improving the appetite:

Cod-liver oil, 19 parts.
Chloral hydrate, cryst, 1 part, mixed.

**Bromide Eruption.**

Dr. Russell reports the case of an epileptic on whom a generalized, painful pustular eruption broke out, while under treatment, by the bromide of potassium. She was at the time taking about four scruples of the bromide per diem. The eruption was at first papular, but soon became pustular. The pustules were all as large as a pea, and some of them as large as two peas; the base was surrounded by an inflammatory zone. When the bromide was stopped the eruption disappeared, but it reappeared again as soon as the medicine was resumed. Dr. Russell at last added five drops of the liquor arsenicalis to each dose of the bromide; in a week the eruption had disappeared and no new pustules were afterwards developed.—*British Med. Jour.*

**Gargle.**

The following, says *Nice Medical*, is much used in Russia and in the Russian colony in Nice:

Carbolic acid, 4 drachms.
Tannin, 4 "
Alcohol, 2 ounces.
Distilled water 4 "

Mix. Dose, a teaspoonful in half a tumblerful of water, to be used once a day. It is found of great benefit in acute angina, and in all chronic irritation of the throat.

**Cold Cream.**

The following will be found the most convenient and easy way of making cold cream, inasmuch as *continuous* stirring is not indispensable:

Lard oil (best), 4 ounces.
White wax, 1 ounce.

Melt; add—

Borax, ½ drachm.

In water, 2 ounces.

Perfume, q. s.

**Bichromate of Potash.**

M. Lanjorrois calls attention to the antiseptic properties of this salt, which appear to be greater than was hitherto supposed. The addition of one per cent. to ordinary water will render it capable of preserving organic substances immersed from all decomposition, even though the solution be exposed to the free contact of the atmosphere. One per cent. is quite sufficient to give a decided tint to water, hence its antiseptic powers seem on a footing with its power of coloration.

**Gout.**

The German papers tell a story of a woman living in the neighborhood of Prague, who suffered so severely from gout in the arm that she could not obtain rest or sleep, and the limb in which the disease had settled was rendered entirely useless. Her husband having heard of a countryman who had been completely cured of rheumatism after being accidentally stung by a bee, persuaded her to try this disagreeable remedy, which, as he pointed out, could hardly prove so painful as the disease. She consented, and allowed three bees to be placed on her arm, and to sting her in several places. Surprising results ensued; the patient soon afterward fell into a long and deep sleep, the first real sleep she had enjoyed for six months, after which the acute pain disappeared, and when the swelling produced by the stings subsided the arm recovered the power of motion, and the gout has not since reappeared.—*Druggists' Circular.*

**Poison-Oak Eruption.**

Dr. Q. C. Smith sends to the *Pacific Medical and Surgical Journal* the following formula for use in this affection:

℞

Acid carbolici, ʒ ss.

Ol. sassafras,

Ol. juniperi, āā ʒ i.

Ung. zinci oxidi (benz.) ℥ i.

M. Apply from two to four times daily, and before going to bed wash the inflamed part thoroughly with warm water and soap.

---

**Nævi.**

Dr. Thiersch applies over the surface of the tumor, a little plate of copper, pierced at regular and short distances with small holes. Through these he passes a needle mounted in a cork, and previously heated in a spirit lamp. The cauterization is thus effected very regularly. The same method is applicable to the linear division of the skin by a cutting needle recently recommended by Mr. Balmanno Squire.— *Med. and Surg. Reporter.*

---

**Salicylic Acid.**

It may be interesting and perhaps useful for some readers of the *Journal* to know that while a solution containing ten grains of salicylic acid and ten grains of borax in one ounce of water has a very bitter taste and an acid reaction, a solution containing ten grains of salicylic acid and fifteen grains of borax, has no disagreeable taste, and is nearly neutral. This solution appears to possess all the valuable properties of salicylic acid, and forms an agreeable means of using the acid internally or as a gargle.—*London Pharm. Jour.*

---

**Soporific.**

In the New York State Inebriate Asylum a glass of milk is frequently administered at bedtime to produce sleep, and the result is often satisfactory, without the use of medicine. Medicine there, is sometimes prescribed in milk. It has been recently stated in the medical journals that *lactic acid* has the effect of promoting sleep by acting as a sedative. As this acid may be produced in the alimentary canal after the ingestion of milk, can this be an explanation of the action of milk on the nervous system when it is "shaky" after a long continued excessive use of alcoholic drink? Sugar, also, is capable of being converted in the stomach, in certain morbid conditions, into lactic acid, and a lump of sugar allowed to dissolve in the mouth on going to bed will frequently soothe a restless body to quiet and repose.

**Carron Oil in Anal Fissure.**

This painful affection, which has heretofore resisted almost all forms of treatment by local applications, has been successfully managed by Carrere, who states in *Annales de la Med. de Gand*, that he applies the mixture of lime and water and linseed oil, so commonly used in burns. This is done several times daily, and in all cases he has obtained a cure in at farthest eight days.—*Allg. Med. Cent.-Zeit.*

---

**Cough Syrup.**

Dr. Kessler says, in *A. M. Jour.*: "We are called frequently in our every-day practice to make up a cough medicine for some of our patients. I have used all the expectorant formulas I have seen, besides a great many of my own mixtures, but so far I have obtained the most flattering results from the following:

℞

Pix liquida, 20 drops.
Spts. nitr. dulc., 1 drachm.
Syr. simpl., 2 ounces.

M. S. Teaspoonful night and morning. Very few doses will suffice in most cases.

---

**Castor Oil Mixture.**

Dr. Wm. V. Ezell, of Carrollton, Ala., sends to the *Louisville Medical News* the following formula, with the statement that it is so palatable that the patient does not recognize the nature of the medicine, unless told what it is:

Castor oil, 1 ounce.
Tincture of cardamon co., 4 drachms.
Oil of wintergreen, 4 drops.
Gum arabic, powdered, 3 drachma.
White sugar, " 2 "
Cinnamon water, to complete, 4 ounces.

Make an emulsion.

---

**Strangulated Hernia.**

The first case was one of recent hernia in the right groin. The tumor was about as large as a chestnut, hard, and too sensitive to permit of the slightest attempt at taxis. The skin over the tumor was washed with warm water containing an alkali, to render absorption more easy, and pure ergotine was then rubbed into it every two hours. At the same time a tablespoonful of a solution consisting of grs. lxxv. of ergotine in ℥ iv. of vehicle was administered every hour. About five hours after the treatment was commenced, the vomiting and colicky pains began to abate, and the tumor became less sensitive, and twelve hours later the hernia returned spontaneously.

In the second case, several unsuccessful attempts had been made to return the hernia by taxis, and leeches and the ether-spray had also been employed in vain. Finally, as the case was becoming desperate, and inflammation was threatening, it was decided to try the ergotine treatment as a last resort before having recourse to operation. The treatment was carried out exactly as in the above case. It was begun at three o'clock in the afternoon, and at six o'clock on the following morning all the symptoms were relieved, and the tumor was reduced with the greatest facility.—*Le Bordeaux Medical.*

---

**Diarrhœa Mixture.**

The following formula is original with Dr. Wm. Gould, of Buffalo, N. Y., where it has proved so efficacious and popular that it is kept in stock by a number of pharmacists:

℞

Tinct. rhei comp., ℥ i.

Tinct. opii, ℥ ss.

Tinct. camphoræ, ʒ ij.

Aq. ammoniæ, ʒ i.

Ol. menth. pip., ʒ ss.

Sig. A teaspoonful in hot sweetened water. Repeat as often as necessary, till relieved.

---

**Mothers' Marks.**

English surgeons have endeavored recently, to remove these frightful disfigurements from the face by surgical treatment. The process is to make parallel incisions the entire thickness of the the skin, with a frozen scalpel, the skin being also frozen by means of the ether spray. The cuts are one-sixteenth of an inch apart, and as soon as these are healed a second set of parallell incisions are made obliquely to the direction of the first set, and so on with a series of operations until complete. Perfect success is the result, as the port wine mark gradually fades away, and is finally obliterated without leaving a scar.

Instruments have since been prepared having sixteen parallel blades, which make the operation very simple to execute.

---

**Wakefulness.**

If you cannot get sleep when you first go to bed, give orders to be waked up at daylight; get up promptly, do not sleep a wink during the day, go to bed at your regular time, with directions to be waked as before ; in a week you will find that you can go to sleep promptly, but then be careful to get up as soon as you wake in the mornings; thus you will soon find out how much sleep your system requires, and act accordingly. Always avoid sleeping in the daytime ; for if you require seven hours' sleep, and spend that much in sleep at night, whatever time you spend in sleep during the day must be deducted from that seven hours, or you will soon be wakeful again. If you wake up in the night, either go to bed two or three hours later, or when you wake, get up, even if it be one o'clock in the morning, and do not sleep a moment until your regular hour for going to bed ; if you wake, and do not sleep in the daytime, you will find out in less than a week how much sleep you require, then act accordingly. Nature loves regularity, and the four hours' sleep from ten to two is said to be worth six hours after twelve o'clock.—*Journal of Health.*

---

**Rheumatism in the Joints.**

When the joints are stiffened with rheumatism or a settled cold, the following applications are capital, and enable the sufferer to move with ease: Cut into small bits (or grate it) one ounce of Castile soap. Add a heaping teaspoonful of red Cayenne pepper. Have these in a small pitcher, and then pour on to them half a pint of boiling water. Stir until all is dissolved, and add a little cider brandy or alcohol when bottling. An application of the above brings the blood in a glow to the joints, and on rubbing a little sweet oil to relax the muscles, the patient will be enabled to walk with ease.—*Druggists Circular.*

---

**Whooping Cough.**

The following formula is recommended by M. Dervieux in the treatment of a commencing pertussis :

℞

Ext. of aconite, gr. $\frac{4}{5}$.

Cherry laurel water, ʒ j.

Syrup of ipecac, ♏ xlv.

Mucilage, ℥ vj.

M.

This is given as soon as the characteristic cough presents itself, in doses of a teaspoonful every hour to young infants, two teaspoonfuls to children over three years of age, and a tablespoonful to adults.—*Hospital Gazette.*

---

**A Preventive of Sea-Sickness.**

Dr. Leaderich claims that collodion has remarkable power as a preventive of sea-sickness. It should be employed as follows: Before embarking, the voyager should apply successively three layers of ricinated collodion to the epigastrium, overstepping slightly the anatomical limits of that region. The layer should extend below to a line immediately above the umbilicus, laterally to a line about two inches outside of the nipples, and above to some inches above the border of the ribs.

When the voyage is expected to last more than a few days, a small quantity of the collodion should be taken along to repair the cracks in the plastering. Dr. Leaderich is unable to say from experience, whether or not the collodion possesses curative as well as preventive powers. He believes it does, however, because he has employed it successfully as an anti-emetic in the painful vomiting of peritonitis. Applied to the epigastrium as above, it always diminished and often arrested the vomiting.—*N. Y. Med. Record, from L'Annee Medicale.*

---

**A Liniment.**

Take half a pound (or less) of bark of wild cherry, and pour on cold water until the bark is well covered. Cover over the bowl and let it remain all night. Next, strain off the liquid, to which add two tablespoonfuls of salt and one tablespoonful of alcohol. When the salt is dissolved pour the liniment in a bottle. The more salt the liquid will take up the better. Weak limbs can be greatly benefited by bathing in this every night.

Another excellent liniment which we have tried is made as follows: Alcohol, 8 ozs.; tincture of aconite, 1 oz.; oil of sassafras, 1 oz.; well rubbed in. It should be marked poison, and kept out of the reach of children.

---

**Rectal Alimentation.**

In the last number of the Circular was published the experience of Dr. Flint and others on this method of administering nourishment. Dr. Armor says in relation to it, that his experience in this way of nourishing patients in extreme cases has been both varied and extensive, and that he has long been satisfied that in cases of necessity it is a most valuable resource. One he lays great stress upon—viz., that whatever substance be selected it must be injected as *tepid fluid and very slowly.* The rectum, like the bladder and other hollow viscera, will not tolerate sudden distention; but with caution on this point it is astonishing how the rectum can be made to tolerate nutritive liquid substances. He has often used the beef-juice mentioned by Dr. Peaslee, but more frequently milk mixed with beef-blood expressed from raw meats and strained. When using stimulants he adds cream to the milk. Small quantities of alcohol are readily tolerated in such a vehicle; and muriate of iron may be administered for a long time in liberal doses, if the bowels be shielded by cream. Rectal medication, as well as alimentation, has not received the attention it deserves. In a large number of chronic diseases, especially when associated with anæmia, in which the stomach for any reason does not tolerate iron, it may be employed by the rectum for a long time with great advantage.

---

**Ergot in Pneumonia.**

J. B. Yeaman, M. D., of Crystal City, Mo. (see *St. Louis Clin. Record*), reports a case of penumonia in a male aged 30, with the physical signs of the first stage well marked; temperature of 104° and a pulse of 108. At noon on the 13th, commenced taking fluid extract of ergot in doses of ʒss. every two hours, the pulse then having risen to 116°, and the temperature to 105.5°. At noon the next day, improvement in every way was marked (muriate of ammonia having also been given ad interim) temperature down to 103°. At 3 P. M. on the 15th, the temperature had fallen 100.5°. By a mistake, the ergot was suspended on the evening of this day, and at 4 P. M. the temperature stood at 101¼°. At 9 A. M. on the fourth day the patient was up and eating breakfast, and was much vexed at being obliged to return to bed. Convalescence was rapid, and on the 20th the patient went out of doors.

---

**Chronic Aural Discharges.**

In a paper read before the Baltimore Academy of Medicine, Dr. Julian J. Chisolm recommends the use of powdered alumen for otorrhœa; he finds it more efficacious than any of the old remedies; he has cured cases of fifty years' standing in one week, and finds very few aural discharges, however chronic, that withstand its proper application.

The method employed in using it, to secure these good results, is first to thoroughly cleanse the ear, then wipe dry the passage by means of a loose cotton swab attached to the end of a match or special applicator; after which, by means of a puff bottle, he puffs into the ear powdered alumen, *filling the drum cavity with it.* The very first application will often indicate a diminished discharge at the end of twenty-four hours. The ear is then washed out, and the alumen powder again applied. This treatment is renewed *once a day* until the discharge is so reduced that the powder blown into the ear continues dry upon its exposed external surface, not enough secretion being formed to permeate the mass of powder. If the powder has crusted in the ear, it may be left for a few days as a hard mass, giving no pain and causing no annoyance. If, after a week or ten days' interval, the ear has seemingly stopped discharging, the alumen powder remaining dry although in a cake, it may be syringed out, as if it were a foreign body. Under these conditions it usually leaves a healthy mucous membrane behind it.

In damp weather he adds to the alumen powder a small quantity of lycopodium, which, when thoroughly triturated with the alumen, makes it more volatile and not at all disposed to lump ; 5 grains of lycopodium powder to the drachm of alumen is ample. In a few cases in which the alumen has failed to stop the aural discharge, he has substituted tannic acid with advantage. With the proper use of these two powders, other local medication in chronic otorrhœa is rarely necessary.

In most cases of ear discharges of long continuance, especially in children of enfeebled constitution, the general medication with cod liver oil and iron tonics must not be omitted.

**Asthma.**

Dr. Wm. M. Welch called the attention of the members to a substance that he had found to be useful in the treatment of asthma. It was a powder to be burned in the sleeping chamber, and the fumes inhaled. It was composed of two and one-half parts of nitrate of potassium, one-half of a part of belladonna, and five of powdered stramonium leaves, intimately mixed with a small proportion, say one-half part, of pulverized white sugar, the latter being added to prevent the compound from burning too freely. The saltpetre may be dissolved in just enough water to make a saturated solution, which is mixed with the leaves, and subsequently the mass dried into a coarse powder, and sugar added. A small quantity is placed on a brick or tin plate and ignited, when it burns, giving off a cloud of smoke. He had occasionally spread sheets over a clothes-horse for the purpose of confining the fumes, and had obtained very satisfactory results during paroxysms of asthma.—*Proceedings of Phil. Co. Med. Soc.*, 1878.

**Boils.**

Dr. Planat, of Nice, claims that arnica has the power of aborting an eruption of boils, except when due to diabetes, with extraordinary rapidity. His method of employing it is very simple. In order to render its action on the small vessels more energetic he applies it directly to the inflamed spot in the form of an ointment, of which the formula is as follows :

Extract of fresh arnica flowers, ʒ ijss.

Honey, ʒ v.

If the mixture be too fluid, he adds powdered lycopodium or althæa, or some other inert powder, until it requires the proper consistency. It is then spread pretty thickly on a bit of oiled silk or diachylon plaster and applied directly to the boil. It is rarely necessary to renew the dressing more than once in twenty-four hours. As a rule, two or three dressings are enough to make the furuncle abort, no matter what may be the period of its evolution.

A curative action is also obtained by the internal administration of the drug. Dr. Planat gives from three to four drops of the tincture, largely diluted, every two hours, and he has seen furuncular eruption disappear very rapidly under the treatment.—*Jour. de Med., etc., de Bruxelles.*

**Danger from the Hypodermic Injection of Morphia.**

In the *Chicago Medical Journal and Examiner*, of May, E. Fletcher Ingalls, of Chicago, gives a supplementary paper on the dangers which may follow the use of morphia hypodermically. He has received a number of letters from various sources confirming the conclusions at which he arrived in his former paper, and says, in brief, as follows :

"I know of no precaution which will render the hypodermic injection of sedative doses of morphia entirely safe ; the medicine may be given in this way a thousand times without harm, but the next time it may be the cause of death. Nothing in the patient's history or general appearance can warn us of the danger, for we find fatal accidents among those who have formerly taken morphia with the happiest results. The danger seems to arise from rapid absorption, or injection directly into the circulation, and it is greatly enhanced by the impossibility of removing the poison.

"All are familiar with the rules laid down for the cautious use of the hypodermic syringe, *i. e.*, to withdraw the point of the syringe slightly after the needle has been introduced as far as desired, to inject the solution slowly, etc. ; but these are not sufficient to insure us against accidents.

"Withdrawing the point of the syringe will not necessarily prevent the solution from passing directly into the circulation ; and as to injecting slowly, probably no one would think of occupying more than five, or at most ten minutes in the operation, which would not be long enough to prevent accidents from rapid absorption. So far as danger is concerned, the hypodermic use of morphia seems to stand in much the same relation to its administration by the stomach, as the inhalation of chloroform does to that of ether ; either method may be fatal, but the latter seems much the safest.

"It is difficult for those who have administered morphia subcutaneously for years, without accident, to realize that there can be danger from the practice; and so it is with chloroform, but we all know that accidents happen from this drug in the hands of the most cautious and skillful surgeons, and considering the large percentage of these fifty-five physicians who have had unpleasant or fatal results from the hypodermic injection of morphia, it would not seem strange if extended and accurate statistics should prove this practice almost if not quite as dangerous as the inhalation of chloroform."

**Ascites Treated by Abdominal Compression.**

Dr. Stephen MacKensie has found compression of the abdomen to be a valuble agent in the treatment of the ascites dependent upon contraction of the liver. The compression accelerates the resorption of the fluid, when this has been initiated by other methods of treatment, such as purgation, deuresis, etc.; more than this, it seems to possess the power of exciting absorption without the intervention of other treatment. Dr. MacKensie records the case of a woman who had suffered for a long time from ascites: She was much emaciated, and œdema of the legs. After some preliminary treatment, a flannel bandage was tightly applied to the abdomen; the pressure at first caused a feeling of sickness, but after a time the bandage ceased to cause discomfort, and appeared to afford relief. It was soon replaced by an elastic abdominal supporter, which was so made that it could be tightened to a considerable degree. The ascites gradually disappeared, and it has not returned, although nearly three years have elapsed since the patient was discharged from the hospital. In a second case the treatment was equally successful, although no internal remedies were used in connection with it. Dr. MacKensie believes that, in spite of the abnormal condition of the liver in these cases, the recovery may be regarded as perfect, since the portions of the organ that remained healthy were sufficient to perform its necessary work. The secondary derangements, due to the mechanical obstruction to the circulation in the liver, were relieved by the diversion of the blood into other channels. The effused fluid is removed from the body through the kidneys, and hence it is essential for the success of the treatment that these organs should be healthy.—*British Med. Jour.*

**On Corns.**

The *Med. and Surg. Reporter* relates that in a lecture at the St. Louis Hospital, Paris, on hypertropy of the epidermis, M. Guibout observed that while in callosities the hypertrophy takes place at the surface, in corns the hypertrophied part becomes pyramidal, and takes the form of a nail, with its point directed toward the deeper-seated parts. This sharp point, lodged in a kind of cupola, which exactly boxes it in, has a tendency to penetrate into the substance of the dermis, whenever the base of the corn is compressed. The portion of the dermis which is in permanent contact with the epidermic induration becomes inflamed and altered in character, its papillæ disappearing, so that at last it becomes a true matrix, destined to form deep, new, horny epidermic layers, in proportion as the more superficial layers are eliminated. Changes of the weather often give rise to great pain in corns, which has been supposed to be due to their hygrometric nature, which, by causing their enlargement, adds to the suffering.. But, in fact, the exacerbations are less servere during the time that it rains, than they are some days preceding; and they are also met with when the weather is about to change from wet to dry. These painful exacerbations of the pain of corns are quite as remarkable and as inexplicable as are those of rheumatic pains. The sole efficacious treatment is excision, but care must be taken that this is complete. The summit of the cone must be cut down too, so as to entirely empty the dermic cupola. And then it is quite necessary to destroy by cauterization the inner surface of this cupola, *i. e.*, the matrix of the corn, which will otherwise be reproduced. The best caustic is sulphuric acid, of which we may deposit a drop, by a match or glass rod, on the excised part. If the corn recurs, the same processes of excision and cauterization must again be resorted to.

**Diseases of the Ear from Bathing.**

Under this heading, Dr. Samuel Sexton, of the New York Ear Dispensary, communicates to *The Medical Record* an elaborate and instructive paper on the dangers of sea-bathing. He attributes many cases of aural disorders and partial or total deafness, to the incautious manner in which many bathers battle with the surf, dive under water, and expose themselves to the cold wind, permitting the hair, ears, etc., to dry by evaporation.

The following conclusions resume the substance of the article, and afford sound advice for sea-bathers:

"The ear is far oftener the seat of inflammation and resulting deafness from bathing than is generally supposed. Although its delicate parts occupy a deep situation in the skull, which is usually a protection from objects capable of injuring it, yet in any bathing which includes immersion of the head, it is liable to be more or less damaged.

"This damage consists in the admission of water to the ear, either through the external auditory canal, or the Eustachian tube.

"When water finds admittance to the former, if cold or salt, inflammation of the meatus alone may result, or if violently injected, as in surf-bathing, or long retained in the canal from diving, the disease may affect the drum-head and middle ear.

"Whenever water is forced from the mouth and nostrils into the middle ear, through the Eustachian tube, inflammation of the middle ear is almost sure to occur, even though the water be warm. This has been frequently illustrated in a most painful manner by those who have been induced to use the popular nasal douche, inflammation of an acute purulent character having frequently been thus established, which has been dangerous to the life of the patient, and after a tedious recovery left great deafness behind.

"Frequent exposures, especially in salt-water bathing, may be the cause of slight earaches, which are usually neglected as unimportant, but which are frequently the precursors of much deafness.

"The fact that several thousand severe cases of aural disease thus result annually in New York City alone, should be a serious admonition to all who are concerned; and that three of the sixty-five cases here reported had dangerous cerebral complications should be a further warning.

"That bathing is pleasant and healthful is of course admitted, but that it cannot be practised as at present without danger is undeniable. It has been shown here by a glance at natural history, that amphibia, whose lives are passed indifferently in either air or water, have naturally the means of protecting their auditory apparatus; but man is not so constructed, and therefore he cannot with safety practice diving or submerging the head.

"He should never dive if he wishes to preserve his hearing.

"When in the surf he should take the water upon his chest or back, closing the mouth and nostrils, being careful not to present the ear to the incoming wave.

"It is equally dangerous while swimming, to receive dashing water into the mouth or nostrils. A firm pledget of cotton-wool in the ears is some protection. Drying the hair and body, and dressing quickly after the bath are, of course, necessary precautions.

"The neglect to observe care in bathing is well illustrated by the four cases of aural disease, which I have presented elsewhere as originating from the Russian bath, two of the attacks happening to physicians. This should point to the necessity of our always putting a patient, advised to use any kind of a bath, upon his guard as regards the danger to the ear.

---

**The Use of Capsicum with Quinia.**

Prof. Wm. H. Thompson says that either capsicum, ginger, or other aromatics, combined with quinia, will diminish the amount required of the latter.

"A good dose of capsicum combined with twenty grains of quinine will act as well as thirty grains of quinine without the capsicum. Spices in general stimulate the portal circulation and promote the flow of bile, and hence their universal use in hot climates. There is tendency on the part of quinine and capsicum to purge, and sometimes to purge violently. In such cases the purgative action is caused by the increased flow of bile produced by the capsicum. Ginger and quinine, when combined, do not purge, and make a very good combination. If the medicine is administered in form of pills, capsicum may be preferable because of the less bulk required; but, if desirable, the ginger may be given separately, and with the same effect as when combined with the quinine. The proportions should be one grain of capsicum to three of quinine; with ginger, one grain of each."—*The Medical Record.*

---

**Whooping-Cough.**

At a recent meeting of the Suffolk District Medical Society, Dr. Pattee called attention to the beneficial effects of Grindelia Robusta in certain pulmonary affections, and remarked that most of the fluid extract sold in this market was said to be worthless. Dr. Pattee had used the tincture in bronchitis, asthma, and whooping-cough, in half a drachm or more, repeated every one or two hours. The effect was said to have been curative in thirty cases of whooping-cough, after three or four days, without the occurence of relapses. The dose for a child two years old would be about ten drops.

A recent telegram from Memphis, giving some particulars regarding the terrible epidemic of yellow fever now raging there, speaks of the fact that but two pharmacies in the city remained open. We would like to publish the names of those men who had the pluck to stay at their posts.

